# Preliminary In Vitro Evaluation of the Growth-Inhibitory Effects of Nigella sativa Oil on Streptococcus mutans

**DOI:** 10.7759/cureus.108151

**Published:** 2026-05-02

**Authors:** Indra Mustapha, Marzia Mustamand, Darryl Caesar, Xinbin Gu

**Affiliations:** 1 Department of Oral Pathology, Howard University, Washington, DC, USA

**Keywords:** antimicrobial, medicine, nigella sativa, oral pathology, pathology, streptococcus mutans

## Abstract

This exploratory study investigated the in vitro growth-inhibitory effects of *Nigella sativa* (*N. sativa*) against *Streptococcus mutans* (*S. mutans*) UA159 in comparison with selected natural products, over-the-counter products (OTC), and reference antimicrobial agents. Agar disk diffusion assays were conducted under anaerobic conditions, and zones of inhibition were analyzed using one-way ANOVA with Dunnett's and Šídák-adjusted post hoc tests. *N. sativa* produced larger inhibition zones than erythromycin (Šídák-adjusted ****p < 0.0001) and chlorhexidine gluconate (Šídák-adjusted ****p < 0.0001) under the tested conditions. A microdilution assay indicated concentration-dependent inhibition of *S. mutans* (0-2.68% v/v), with an estimated IC₅₀ of 1.45% (v/v). These findings indicate that *N. sativa* exhibits measurable in vitro growth-inhibitory effects, supporting further investigation to more fully characterize its antimicrobial potential.

## Introduction

*Nigella sativa* (*N. sativa*), commonly known as black seed or black cumin, has been valued for its medicinal properties throughout history [1]. It has been widely used in traditional medicine and has demonstrated beneficial effects in the treatment of cardiovascular, infectious, and inflammatory diseases [1]. Thymoquinone (TQ) is considered the principal bioactive constituent of *N. sativa* [1]. In particular, the seeds of *N. sativa* are widely used in the treatment of hypertension, diabetes, respiratory and gastrointestinal conditions, owing in part to their well-documented antibacterial and anti-inflammatory properties [1]. In vitro studies using a water-based extract of *N. sativa* seeds, designed to simulate oral exposure during chewing, demonstrated significant cytotoxic effects on oral cancer and pre-cancerous leukoplakia cells; this activity was associated with higher levels of the water-soluble constituent α-hederin, while thymoquinone was detected only in modest amounts, suggesting limited release in this aqueous model [2]. 

Quite notably,* N. sativa* has garnered attention for its reported antimicrobial and antibiofilm effects, with proposed mechanisms including disruption of extracellular polysaccharide synthesis and inhibiting enzymes such as glucosyltransferase, thereby destabilizing cariogenic biofilms [3-5]. These properties are particularly significant as they target biofilm-associated microbes involved in chronic oral infections while also exerting anti-inflammatory and immunoregulatory effects that support periodontal tissue healing and enhance overall therapeutic potential [3, 6]. Moreover, *N. sativa* is now being incorporated into the oral care market, called SprinJene, as an implementation in a toothpaste formulation [7].

*Streptococcus mutans *(*S. mutans*)* *is a Gram‑positive, facultative anaerobic coccus that produces acid in the oral biofilm and is strongly associated with caries history; its prevalence and abundance are consistently higher in individuals with caries compared with caries‑free controls in clinical microbiome studies [8, 9]. Emerging research shows that *S. mutans* can enter the bloodstream from oral lesions, and strains expressing collagen‑binding surface proteins have been linked to endothelial invasion and inflammation, with *S. mutans* bacteremia reported in infective endocarditis cases [10]. Recent studies have shown that *S. mutans* growth and biofilm formation can be inhibited by *N. sativa *extracts, while erythromycin has long demonstrated antibacterial activity against *S. mutans*, highlighting their potential as antimicrobial or adjunctive agents [11-13]. Chlorhexidine remains one of the most studied antimicrobials against *S. mutans*, effectively reducing its growth and biofilm formation [14]. Essential oils like eucalyptus, cinnamon, and thyme have demonstrated significant antibacterial and antibiofilm activity against *S. mutans* in vitro, with eucalyptus oil reducing planktonic and biofilm growth, and thyme/cinnamon oils showing strong inhibitory effects that suggest potential as adjunctive or alternative antimicrobial agents [14, 15].

With the literature demonstrating the antimicrobial and antibiofilm activity of these agents against *S. mutans*, this preliminary screening study aimed to evaluate the in vitro growth-inhibitory effects of *N. sativa*, eucalyptus oil, cinnamon oil, thyme oil, erythromycin, chlorhexidine, and SprinJene, against *S. mutans* using a zone of inhibition assay on agar, followed by the determination of the estimated dose-dependent inhibition percentage of* N. sativa*.

SprinJene is a commercially available oral care formulation with a proprietary ingredient profile, which limits the ability to attribute observed biological effects to individual components and necessitates cautious interpretation of comparative findings. Furthermore, it is important to emphasize that this study constitutes an in vitro screening investigation aimed at identifying potential growth-inhibitory effects of *N. sativa* and does not represent a standardized antimicrobial susceptibility assessment.

## Materials and methods

Zone of inhibition assays: bacterial strain, culture conditions, preparation of selective media, preparation of test agents, data collection, and statistical analyses

*S. mutans* UA159 (serotype c) was obtained commercially. The organism was cultured in Brain Heart Infusion (BHI) broth at 37°C for 72 hours under anaerobic conditions (5% nitrogen, 1.7% hydrogen). *S. mutans* UA159 was selected as a well-characterized reference strain widely used in oral microbiology research. As this study was designed as a preliminary in vitro evaluation, a single standardized strain was used to ensure reproducibility and minimize experimental variability.

Selective agar plates were prepared using Mitis Salivarius Bacitracin agar (MSBA) according to the manufacturer's instructions. Upon cooling of the sterilized medium, 1% potassium tellurite was added. The medium was then poured into sterile Petri dishes and allowed to solidify at room temperature. Mitis Salivarius Bacitracin agar (MSBA) was selected to provide selective growth conditions for *S. mutans*. As this study was designed as a preliminary in vitro evaluation rather than a standardized antimicrobial susceptibility assay, Mueller-Hinton agar was not used.

Following incubation, the bacterial suspension was subjected to agar diffusion analysis. Briefly, 100 µL of the overnight culture of *S. mutans* UA159 was evenly streaked onto selective Mitis Salivarius Bacitracin agar plates to obtain a uniform lawn of growth. Sterile 5-mm diameter filter paper discs were impregnated with 20 µL of each antimicrobial agent and aseptically placed onto the inoculated agar surface. Each agent was tested in duplicate per experiment, and a total of five independent experiments were performed to ensure reproducibility.

All essential oils (*N. sativa*, cinnamon, eucalyptus, and thyme) were commercial-grade preparations, and their specific chemical compositions were not analytically characterized. Essential oils were tested at 100% (v/v) concentration to evaluate maximal inhibitory activity in this in vitro screening model and to characterize intrinsic growth-inhibitory effects under non-diluted conditions.

A SprinJene toothpaste slurry was prepared at a 1 g toothpaste per 1 mL sterile distilled water (100% w/v) ratio. Sterile distilled water served as the negative control, and 100% (v/v) ethanol served as the vehicle control. Erythromycin (10 mg/mL prepared in 100% ethanol) and chlorhexidine gluconate (0.12% v/v) were included as positive controls, representing an antibiotic and an oral antiseptic benchmark, respectively.

Plates were incubated at 37°C for 72 hours under anaerobic conditions (5% nitrogen and 1.7% hydrogen). After incubation, the radius of the zone of inhibition was measured in millimeters (mm) from the edge of the disc to the edge of the bacterial growth using a digital caliper. Samples were assigned coded identifiers prior to analysis, and zone of inhibition measurements were performed by an investigator blinded to treatment allocation.

Statistical analysis

Normality of data distribution was assessed using the Shapiro-Wilk test. As all treatment groups exhibited normal distributions, differences among groups were analyzed using one-way analysis of variance (ANOVA). Dunnett's multiple comparisons test was used to compare each treatment group with the water control. A priori planned pairwise comparisons between selected natural products (*N. sativa*, thyme oil, cinnamon oil), an over-the-counter formulation (SprinJene), and reference antimicrobial agents (erythromycin and chlorhexidine gluconate) were performed using Šídák-adjusted tests. Effect sizes (Cohen's d) were calculated for all pairwise comparisons as standardized mean differences using pooled standard deviations derived from the dataset. Values were computed as the mean difference divided by the pooled standard deviation. The sign of *d* reflects the direction of the comparison (water control minus treatment), while the magnitude reflects the strength of the effect.

Data are presented as mean ± standard deviation (SD). Statistical significance was set atp < 0.05. For statistically significant comparisons, exact *p*-values were below the numerical resolution of GraphPad Prism and are therefore reported as p< 0.0001, consistent with standard reporting conventions.

Dose-dependent microdilution assay

A fresh culture of *S. mutans* UA159 was prepared by inoculating 20 mL of Tryptic soy broth (TSB) with the stock culture and incubating at 37°C for 48 h under anaerobic conditions. The culture was diluted 1:50 in fresh TSB to standardize the inoculum. Aliquots (200 µL) of the diluted bacterial suspension were dispensed into wells of a sterile 96-well microtiter plate.

*N. sativa* oil was added directly to designated wells to achieve final concentrations of 0%, 0.1%, 0.2%, 0.398%, 0.695%, 1.36%, and 2.68% (v/v), calculated relative to total well volume. Control wells contained bacterial suspension without oil (0% v/v).

Plates were incubated at 37°C for 24 hours under anaerobic conditions. Bacterial growth was quantified by measuring optical density at 585 nm using the FilterMax™ F5 Multi-Mode Microplate Reader (Molecular Devices, San Jose, California). Absorbance values were blank corrected using uninoculated broth and normalized to untreated controls. Each concentration was tested in triplicate wells per experiment, and experiments were performed independently three times. Data is expressed as mean values.

## Results

Comparative antibacterial efficacy of natural products and standard antimicrobials against *Streptococcus mutans*

One-way ANOVA demonstrated a significant overall effect of treatment on zone of inhibition (F(8, 90) = 756.3, p < 0.0001), with a very large effect size (η² = 0.985), indicating that the majority of variability in inhibition zones was attributable to treatment differences. Dunnett's multiple comparisons test showed that all treatments except eucalyptus oil produced significantly larger zones of inhibition than the water control (all Dunnett-adjusted p < 0.0001). In contrast, eucalyptus oil did not differ significantly (Dunnett-adjusted p = 0.1160) (Table 1). 

**Table 1:** Dunnett's multiple comparisons test comparing zones of inhibition for each treatment group relative to the water control Data are presented as mean differences (mm), 95% confidence intervals (CI), and Dunnett-adjustedp-values from one-way ANOVA. Effect sizes (Cohen's d) represent standardized mean differences between groups. NS - *N. sativa*; T - thyme oil; C - cinnamon oil; Er - erythromycin; S - SprinJene toothpaste; Ch - chlorhexidine gluconate; E - Eucalyptus oil; Et - ethanol; W - water

Comparison	Mean diff (mm)	95% CI	Dunnett-adjusted p-value	Effect Sizes (Cohen's d)
W vs NS	-25.50	-27.00 to -23.99	<0.0001	-13.8
W vs T	-24.62	-26.12 to -23.11	<0.0001	-13.3
W vs C	-19.38	-20.88 to -17.87	<0.0001	-10.5
W vs S	-7.017	-8.52 to -5.51	<0.0001	-3.8
W vs Er	-18.76	-20.26 to -17.25	<0.0001	-10.1
W vs Ch	-3.397	-4.90 to -1.89	<0.0001	-1.8
W vs E	-1.309	-2.81 to 0.19	0.1160	-0.7
W vs Et	0.00	-1.50 to 1.50	>0.9999	0.00

For all statistically significant comparisons, exact *p*-values were below the reporting threshold of the statistical software and are therefore reported as p < 0.0001. 

**Figure 1:**
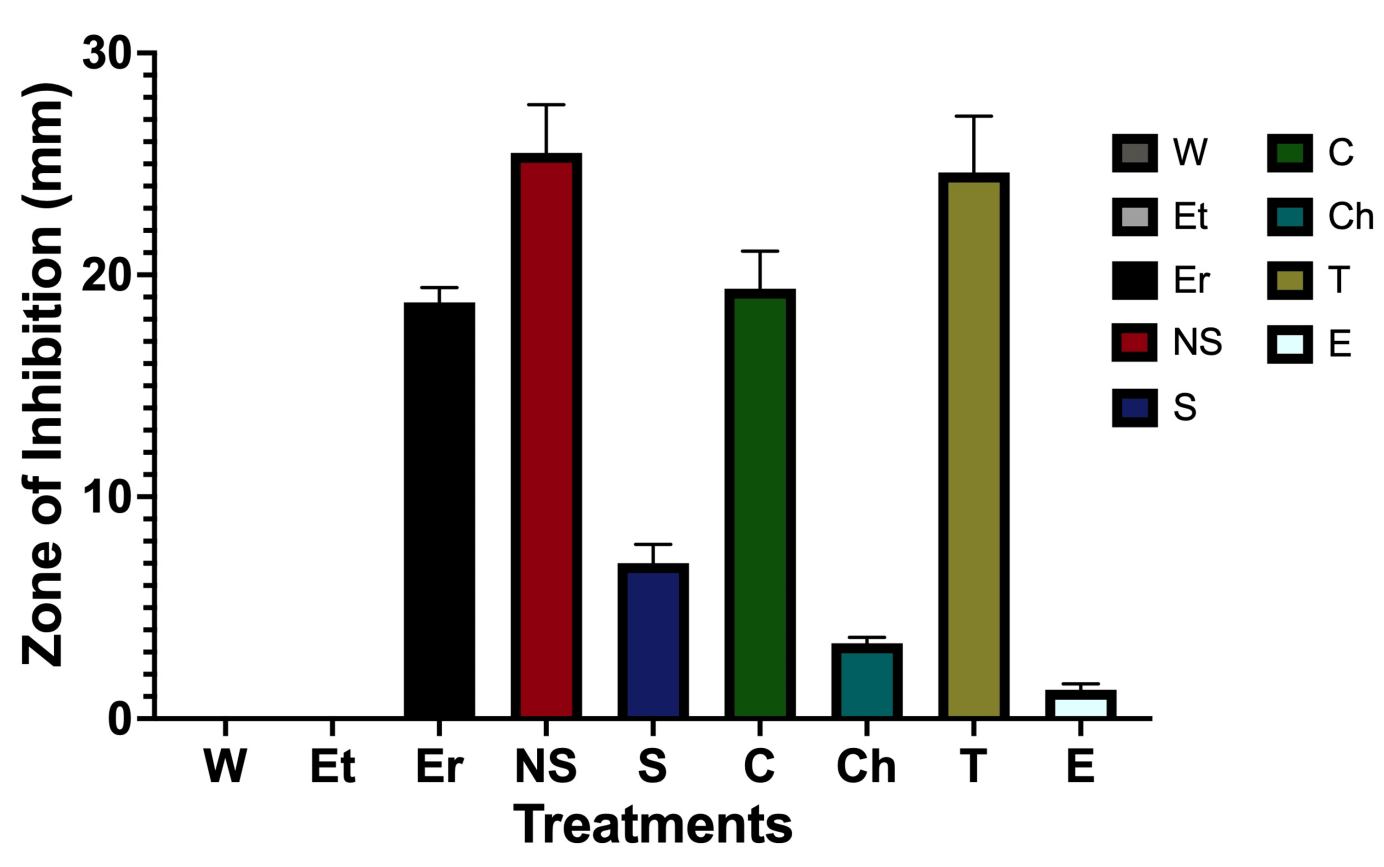
Zones of inhibition (mm) of tested natural products and clinical antimicrobials against Streptococcus mutans UA15 Data represent mean ± SD. NS, T, C, S, Er, and Ch demonstrated larger inhibition zones than water under the conditions tested (Dunnett's test, ****p < 0.0001), whereas Et and E were not significant (ns). NS - N. sativa; T - thyme oil; C - cinnamon oil; Er - erythromycin; S - SprinJene toothpaste; Ch - chlorhexidine gluconate; E - Eucalyptus oil; Et - ethanol; W - water

As shown in Figure 1, *N. sativa *(NS) and thyme oil (T) produced the largest zones of inhibition against *S. mutans*, followed by cinnamon oil (C), erythromycin (Er), and SprinJene toothpaste (S). Chlorhexidine gluconate showed comparatively smaller inhibition zones, while eucalyptus oil exhibited minimal activity. Ethanol and water controls produced no detectable inhibition. 

Planned a priori pairwise comparisons using Šídák's multiple comparisons test showed that *N. sativa* produced larger inhibition zones than erythromycin under the conditions tested (mean difference = 6.73 mm, 95% CI 5.32-8.15, Šídák-adjusted p < 0.0001) and chlorhexidine gluconate (mean difference = 22.10 mm, 95% CI 20.69-23.51, Šídák-adjusted p< 0.0001). Thyme oil also exhibited larger inhibition zones than erythromycin under the conditions tested (mean difference = 5.86 mm, 95% CI 4.44-7.27, Šídák-adjusted p < 0.0001), whereas cinnamon oil did not differ significantly (ns) from erythromycin (Šídák-adjusted p= 0.72). The commercially available toothpaste, SprinJene, produced a larger zone of inhibition compared with the product, chlorhexidine gluconate (mean difference, 3.62 mm; 95% CI, 2.52-4.73 mm; Šídák-adjusted p < 0.0001). These planned comparisons are summarized in Table 2.

**Table 2:** Planned a priori pairwise comparisons between selected natural products, an OTC formulation, and reference antimicrobial agents Values are presented as mean differences (mm), 95% confidence intervals (CI), Šídák-adjusted *p*-values, and effect sizes (Cohen's d). NS - N. sativa; T - thyme oil; C - cinnamon oil; Er - erythromycin; S - SprinJene toothpaste; Ch - chlorhexidine gluconate; E - Eucalyptus oil; Et - ethanol; W - water; OTC - over-the-counter products

Comparison	Mean diff (mm)	95% CI	Šídák-adjusted p-value	Effect Size (Cohen's d)
NS vs Er	6.73	5.32–8.15	<0.0001	5.16
NS vs Ch	22.10	20.69–23.51	<0.0001	16.93
C vs Er	0.62	-0.80–2.03	0.72	0.47
T vs Er	5.86	4.44–7.27	<0.0001	4.49
S vs Ch	3.62	2.52 to 4.73	<0.0001	2.77

While these findings demonstrate clear differences in zone formation under the experimental conditions, they should be interpreted with caution and in the context of an exploratory screening study. Agar diffusion assays are influenced by compound solubility, molecular size, and diffusion characteristics; therefore, zone diameters do not directly equate to antimicrobial potency.

Dose-response effect of *Nigella sativa* on *Streptococcus mutans* growth

To further characterize the growth-inhibitory effects of *N. sativa*, a dose-response analysis was performed using concentrations of 0%, 0.1%, 0.2%, 0.398%, 0.695%, 1.36%, and 2.68% (v/v). Bacterial growth was quantified by measuring optical density at 585 nm (OD₅₈₅). Optical density at 585 nm (OD₅₈₅) was normalized to the untreated control (0% NS), which was set to 100%, and expressed as a percentage of the control.

**Figure 2:**
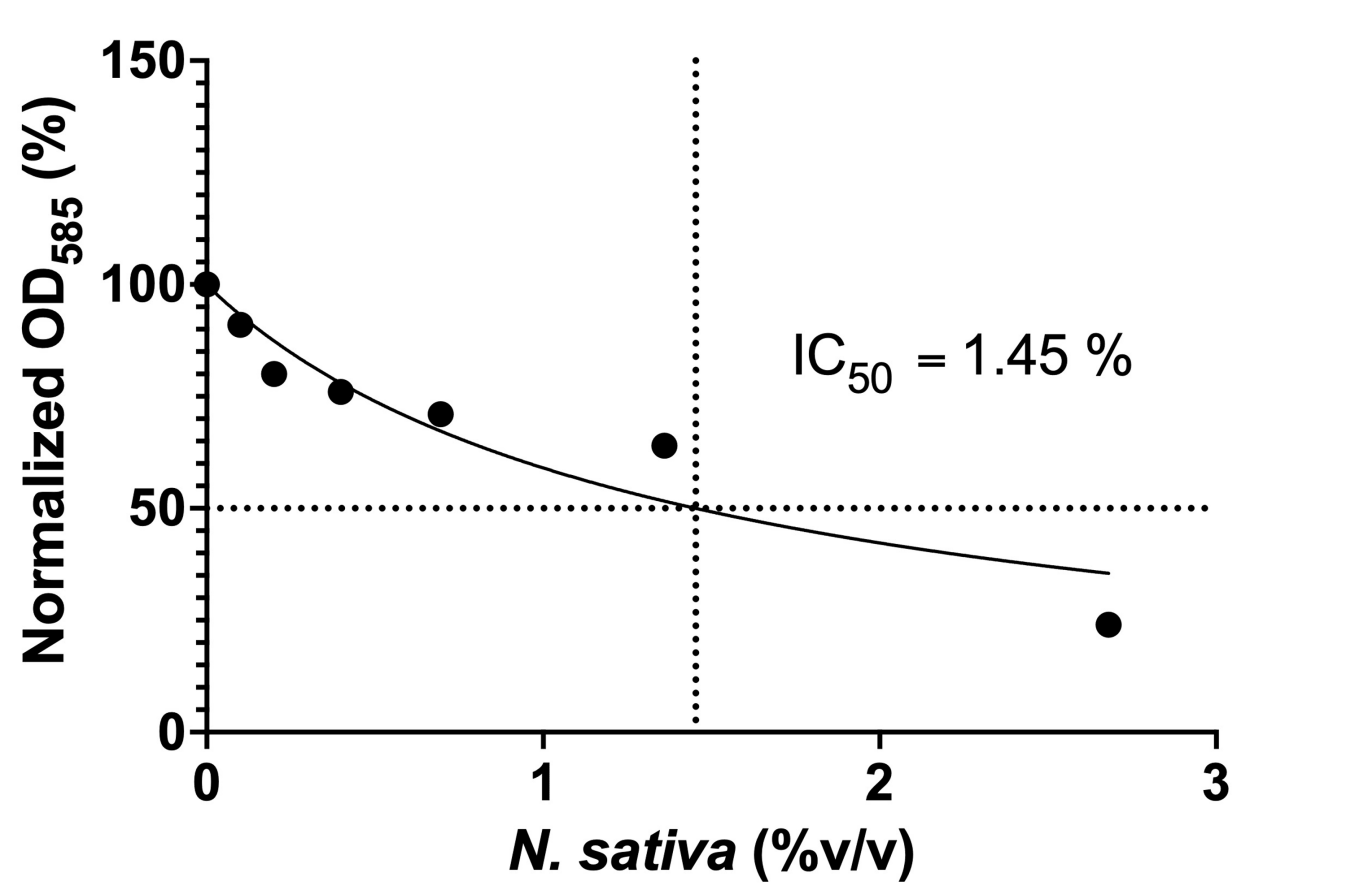
Dose-response effect of Nigella sativa on Streptococcus mutans growth Normalized OD585 (%) of *S. mutans* following treatment with increasing concentrations of *Nigella sativa* (0–2.68% v/v). Data were fitted using nonlinear regression (variable-slope model). Bacterial growth decreased in a concentration-dependent manner, with an estimated IC₅₀ = 1.45% (v/v)

As shown in Figure 2, increasing concentrations of *N. sativa *resulted in a progressive reduction in OD₅₈₅ expressed as a percentage of the control, indicating concentration-dependent inhibition of *S. mutans*. Minimal reduction in bacterial growth was observed at 0.1% and 0.2% relative to the untreated control (0%). Inhibition was observed between 0.398% and 0.695%, whereas more pronounced suppression of growth occurred at 1.36% and 2.68%.

Nonlinear regression analysis using a variable-slope dose-response model with an estimated half-maximal inhibitory concentration (IC₅₀) of 1.45% (v/v) (95% CI: 0.90-3.23). These findings demonstrate a concentration-dependent growth-inhibitory effect of *N. sativa *against *S. mutans*, consistent with the inhibitory activity observed in the disk diffusion assay.

## Discussion

This exploratory investigation suggests that *N. sativa *exhibits measurable growth-inhibitory effects in vitro against *S. mutans *under the experimental conditions tested, as demonstrated by larger zones of inhibition in disk diffusion assays and a measurable concentration-dependent reduction in bacterial growth. Comparative analyses further indicated that *N. sativa* produced larger inhibition zones than erythromycin and chlorhexidine under the conditions tested. The estimated half-maximal inhibitory concentration (IC₅₀) of 1.45% (v/v) provides a model-derived estimate of inhibitory concentration within the tested range. Although limited in scope, these findings suggest that *N. sativa* possesses bioactive properties warranting further investigation as a potential alternative or adjunct antimicrobial agent.

Interest in plant-derived antimicrobials has increased in response to rising antibiotic resistance and the need for novel therapeutic strategies [16, 17]. Recent studies have reported that extracts and essential oil components of *N. sativa*, particularly thymoquinone, exhibit antibacterial activity against Gram-positive organisms, including oral streptococci [18, 19]. These compounds are proposed to disrupt membrane integrity, interfere with metabolic pathways, and impair virulence-associated processes [20]. While mechanistic evaluation was beyond the scope of the present study, the dose-dependent inhibition observed here aligns with these emerging findings and supports continued pharmacologic characterization.

Notably, *N. sativa* is listed as an ingredient in the commercially available oral care product SprinJene; however, the concentration of *N. sativa* oil in this formulation is undisclosed, and its specific antimicrobial contribution has not been independently evaluated [7]. In our experimental model, this OTC formulation produced larger inhibition zones than chlorhexidine gluconate under the experimental conditions. However, given the proprietary formulation of SprinJene, the relative contribution of individual ingredients to the observed antimicrobial activity cannot be ascertained from the present data.

Diffusion assays should be interpreted cautiously; essential oils were evaluated at full strength in agar diffusion assays, and differences in molecular weight, volatility, and hydrophobicity may have influenced zone diameters independently of intrinsic antimicrobial potency. Agar-based methods reflect both antimicrobial potency and compound diffusion characteristics, which may favor certain hydrophobic agents [21]. Moreover, in vitro growth inhibition does not directly translate to clinical efficacy, particularly for infections involving structured biofilms such as plaque [22]. Therefore, these data should be viewed as early-stage screening results rather than evidence of therapeutic equivalence to established antibiotics. 

While the antimicrobial activity of *N. sativa* has been previously reported, this study provides a quantitative evaluation of its growth-inhibitory effects against *S. mutans* using a dose-response approach and model-derived IC₅₀ estimation. In addition, the inclusion of multiple natural products, conventional antimicrobial agents, and a commercially available oral care formulation within a single experimental framework offers a comparative context that is not commonly addressed in prior studies. Although these findings should not be interpreted as direct indicators of clinical efficacy, they contribute to a more structured baseline for future mechanistic, translational, and standardized investigations.

Limitations and future directions

These findings should be interpreted within the limitations of this exploratory screening study, including the use of a single bacterial strain, agar diffusion-based susceptibility testing, and the absence of standardized MIC and MBC determinations. Differences in vehicle conditions across test agents due to formulation variability (including ethanol-based solubilization, aqueous preparations, and undiluted formulations) may have influenced comparative inhibition zone measurements, alongside inherent variability in diffusion characteristics and physicochemical properties of the tested agents. Formal randomization of treatment allocation was not performed, which may introduce potential selection bias; however, outcome assessment was conducted in a blinded manner to reduce measurement bias. Furthermore, the essential oils were used as commercially available preparations without analytical characterization of their chemical composition, and thus batch-to-batch or product-specific variability cannot be excluded; future studies employing gas chromatography-mass spectrometry (GC-MS) analysis are warranted to characterize phytochemical profiles and better relate active constituents to antimicrobial effects. 

The growth-inhibitory effects observed in this preliminary study support further investigation, including standardized antimicrobial assays, to more precisely characterize the activity of* N. sativa*. Although a formal minimum inhibitory concentration (MIC) was not determined in the present exploratory study, the dose-response modeling provides an initial quantitative estimate of inhibitory concentration ranges. Future studies should include MIC and minimum bactericidal concentration (MBC) determinations, mechanism-of-action analyses, evaluation against resistant strains, and assessment in clinically relevant biofilm models. Collectively, these findings contribute to the growing body of literature exploring plant-derived compounds as potential candidates in the development of novel antimicrobial strategies.

## Conclusions

This exploratory in vitro investigation indicates that *N. sativa* exhibits measurable growth-inhibitory activity against* S. mutans* UA159, as demonstrated by significant zones of inhibition and a concentration-dependent reduction in bacterial growth. Dose-response analysis identified an estimated IC₅₀ of 1.45% (v/v), suggesting a quantifiable inhibitory effect within the tested experimental range. In comparative agar diffusion analyses, *N. sativa* produced larger zones of inhibition than erythromycin and chlorhexidine gluconate under the conditions tested. These findings are specific to the model employed and are influenced by compound diffusion characteristics and assay conditions. Overall, these findings demonstrate that *N. sativa *exhibits in vitro growth-inhibitory activity against *S. mutans* UA159, supporting its potential role in the development of novel antimicrobial strategies in relation to oral health applications.

## References

[1] Alberts A, Moldoveanu ET, Niculescu AG, Grumezescu AM (2024). Nigella sativa: A comprehensive review of its therapeutic potential, pharmacological properties, and clinical applications. Int J Mol Sci.

[2] Dagtas S, Griffin RJ (2021). Nigella sativa extract kills pre-malignant and malignant oral squamous cell carcinoma cells. J Herb Med.

[3] Tucakov L, Tomić A, Šovljanski O, Aćimović M, Miljković A (2025). Targeting oral pathogens with salvia officinalis and nigella sativa supercritical CO2 extracts: A pharmacodynamic approach and three-dimensional checkerboard synergy for novel dental antimicrobials. Antibiotics (Basel).

[4] Zeineldin M, Esmael A, Al-Hindi RR, Alharbi MG, Ashenafi Bekele D, Teklemariam AD (2023). Beyond the risk of biofilms: an up-and-coming battleground of bacterial life and potential antibiofilm agents. Life (Basel).

[5] Mulat M, Banicod RJ, Tabassum N (2025). Multiple strategies for the application of medicinal plant-derived bioactive compounds in controlling microbial biofilm and virulence properties. Antibiotics (Basel).

[6] Majdalawieh AF, Fayyad MW (2015). Immunomodulatory and anti-inflammatory action of Nigella sativa and thymoquinone: a comprehensive review. Int Immunopharmacol.

[7] (2026). SprinJene natural total care fluoride-free toothpaste. https://sprinjene.com/products/sprinjene-natural-total-care-fluoride-free-toothpaste..

[8] Mazurel D, Brandt BW, Boomsma M, Crielaard W, Lagerweij M, Exterkate RA, Deng DM (2025). Streptococcus mutans and Caries: a systematic review and meta-analysis. J Dent Res.

[9] Lemos JA, Quivey RG, Koo H, Abranches J (2013). Streptococcus mutans: a new Gram-positive paradigm?. Microbiol.

[10] Xiao H, Li Y (2025). From teeth to body: the complex role of Streptococcus mutans in systemic diseases. Mol Oral Microbiol.

[11] Kamali SA, Rezvani MB, Pourhajibagher M, Farzaneh F, Emami-Razavi HS (2024). In vitro synergistic antibacterial effects of extract and honey derived from Nigella sativa on Streptococcus mutans: antibacterial effects of Nigella sativa extract and honey. Galen Med J.

[12] Jassim Mohammed Z, Mohammad Hussein J (2025). Efficacy of some antibiotics against Streptococcus mutans associated with periodontitis in patients. MID.

[13] Little WA, Thomson LA, Bowen WH (1979). Antibiotic susceptibility of Streptococcus mutans: comparison of serotype profiles. Antimicrob Agents Chemother.

[14] de Oliveira Carvalho I, Purgato GA, Píccolo MS, Pizziolo VR, Coelho RR, Diaz-Muñoz G, Alves Nogueira Diaz M (2020). In vitro anticariogenic and antibiofilm activities of toothpastes formulated with essential oils. Arch Oral Biol.

[15] Balhaddad AA, AlSheikh RN (2023). Effect of eucalyptus oil on Streptococcus mutans and Enterococcus faecalis growth. BDJ Open.

[16] (2023). World Health Organization. Global antimicrobial resistance and use surveillance system (GLASS) report. https://www.who.int/publications/i/item/9789240062702.

[17] Murray CJL, Ikuta KS, Sharara F (2022). Global burden of bacterial antimicrobial resistance in 2019: a systematic analysis. Lancet.

[18] Chatterjee G, Saha AK, Khurshid S, Saha A (2025). A comprehensive review of the antioxidant, antimicrobial, and therapeutic efficacies of black cumin (Nigella sativa L.) seed oil and its thymoquinone. J Med Food.

[19] Ahmad A, Husain A, Mujeeb M (2013). A review on therapeutic potential of Nigella sativa: a miracle herb. Asian Pac J Trop Biomed.

[20] Kashi M, Varseh M, Hariri Y, Chegini Z, Shariati A (2025). Natural compounds: new therapeutic approach for inhibition of Streptococcus mutans and dental caries. Front Pharmacol.

[21] Balouiri M, Sadiki M, Ibnsouda SK (2016). Methods for in vitro evaluating antimicrobial activity: a review. J Pharm Anal.

[22] Koo H, Allan RN, Howlin RP, Stoodley P, Hall-Stoodley L (2017). Targeting microbial biofilms: current and prospective therapeutic strategies. Nat Rev Microbiol.

